# Nogo-A Induced Polymerization of Microtubule Is Involved in the Inflammatory Heat Hyperalgesia in Rat Dorsal Root Ganglion Neurons

**DOI:** 10.3390/ijms221910360

**Published:** 2021-09-26

**Authors:** Ling Chen, Qiguo Hu, Huaicun Liu, Yan Zhao, Sun-On Chan, Jun Wang

**Affiliations:** 1 Department of Human Anatomy, Histology & Embryology, School of Basic Medical Sciences, Peking University, Beijing 100191, China; cl2010sbh@163.com (L.C.); 18801235251@163.com (Q.H.); liuhc@bjmu.edu.cn (H.L.); zhaoyan0118@pku.edu.cn (Y.Z.); 2 School of Biomedical Sciences, Faculty of Medicine, The Chinese University of Hong Kong, Hong Kong, China

**Keywords:** microtubule, Nogo-A, inflammatory heat hyperalgesia, DRG, rat

## Abstract

The microtubule, a major constituent of cytoskeletons, was shown to bind and interact with transient receptor potential vanilloid subfamily member 1 (TRPV1), and serves a pivotal role to produce thermal hyperalgesia in inflammatory pain. Nogo-A is a modulator of microtubule assembly and plays a key role in maintaining the function of TRPV1 in inflammatory heat pain. However, whether the microtubule dynamics modulated by Nogo-A in dorsal root ganglion (DRG) neurons participate in the inflammatory pain is not elucidated. Here we reported that the polymerization of microtubules in the DRG neurons, as indicated by the acetylated α-tubulin, tubulin polymerization-promoting protein 3 (TPPP3), and microtubule numbers, was significantly elevated in the complete Freund’s adjuvant (CFA) induced inflammatory pain. Consistent with our previous results, knock-out (KO) of Nogo-A protein significantly attenuated the heat hyperalgesia 72 h after CFA injection and decreased the microtubule polymerization via up-regulation of phosphorylation of collapsin response mediator protein 2 (CRMP2) in DRG. The colocalization of acetylated α-tubulin and TRPV1 in DRG neurons was also reduced dramatically in Nogo-A KO rats under inflammatory pain. Moreover, the down-regulation of TRPV1 in DRG of Nogo-A KO rats after injection of CFA was reversed by intrathecal injection of paclitaxel, a microtubule stabilizer. Furthermore, intrathecal injection of nocodazole (a microtubule disruptor) attenuated significantly the CFA-induced inflammatory heat hyperalgesia and the mechanical pain in a rat model of spared nerve injury (SNI). In these SNI cases, the Nogo-A and acetylated α-tubulin in DRG were also significantly up-regulated. We conclude that the polymerization of microtubules promoted by Nogo-A in DRG contributes to the development of inflammatory heat hyperalgesia mediated by TRPV1.

## 1. Introduction

Inflammatory factors from damaged tissues lower the threshold of nociceptors on nerve fibers and cause inflammatory pain [1]. This pain sensation is exuberated in inflammatory thermal hyperalgesia, which is largely mediated by the transient receptor potential vanilloid subfamily member 1 (TRPV1) in primary sensory neurons [2,3]. Further studies show that the activation of TRPV1 is under the regulation of the cytoskeleton system [4,5,6].

The cytoskeleton consists of filament, microtubule, and intermediate fiber [7]. The microtubule is one of the major cytoskeletal components of neurons [8]. It is built from heterodimers of α- and β-tubulin [9] and is polymerized and depolymerized dynamically [10]. The abnormality of tubulin modification was shown to underlie the pathogenesis of Parkinson’s disease. The reduction of Tyr-α-tubulin and enrichment of Ace-α-tubulin in microtubules are early events specifically associated with the degeneration of dopaminergic neurons [11]. Microtubule abnormality is also reported to be involved in Alzheimer’s disease, amyotrophic lateral sclerosis, and traumatic brain injury [12,13,14,15].

Several studies have suggested the important roles of microtubules in pain modulation. The acetylated microtubules, an indicator of the polymerized microtubules, are necessary for mechanical touch and pain in mice [16,17,18]. Moreover, it was reported that the microtubule can interact with and regulate the function of TRPV1 [19,20]. Earlier studies indicated the coexistence of TRPV1 complex on microtubules in nerve endings and neurons [21,22]. However, the exact function of microtubules in pain modulation is still elusive. Microtubule-disrupting drugs, nocodazole, colcemid, and vincristine, attenuate significantly epinephrine (EPI) induced hyperalgesia but show no effect on prostaglandin-E2 (PGE2) induced hyperalgesia [4]. Our previous study demonstrated that Nogo-A, a cytoskeleton modulator, promotes inflammatory heat hyperalgesia via maintaining the function of TRPV1 in DRG neurons [5]. It remains to be determined whether Nogo-A works as an upstream modulator of microtubules to develop inflammatory pain.

In the adult central nervous system, Nogo-A inhibits axonal regeneration after nerve injury. It also inhibits the plasticity of synapses and so stabilizes the neural circuits [23]. As an important cytoskeletal regulator, Nogo-A regulates various cytoskeletal components such as microtubules, microfilaments, and motor proteins [24,25,26]. It plays a crucial role in regulating cerebral hemorrhage, amyotrophic lateral sclerosis, senile dementia, and other diseases [27]. Recent reports have shown that Nogo participates in different pathological processes via modulating the microtubules. For example, knockdown of RTN4 (Nogo) destabilizes tubulins and prompts paclitaxel-induced cytotoxicity in cancers [28]. Nogo may act through the collapsing response mediator protein-2 (CRMP2) [26,29], which binds to tubulin heterodimers and promotes microtubule assembly [30]. Phosphorylation of CRMP2 decreases the active form of CRMP-2, and so impairs neuronal polarity by suppressing axon elongation [31]. These findings suggest that Nogo-A is an important upstream modulator of the CRMP2/tubulin pathway. Taking together that Nogo-A in DRG neurons contributes to the inflammatory pain via maintaining the function of TRPV1 [5], it is hypothesized that the Nogo-A/CRMP2/tubulin pathway is involved in pain modulation. 

In this study, we provide evidence that polymerization of microtubules is increased significantly in DRG neurons in CFA-induced inflammatory pain. Nogo-A in DRG neurons promotes the polymerization of microtubules via inhibiting phosphorylation of CRMP2. The polymerization of tubulin prompted by Nogo-A is necessary to maintain the expression of TRPV1 and to develop the CFA-induced inflammatory heat hyperalgesia.

## 2. Results

### 2.1. Polymerization of Microtubule in DRG Neurons Was Increased in CFA-Induced Inflammatory Pain 

**Figure 1:**
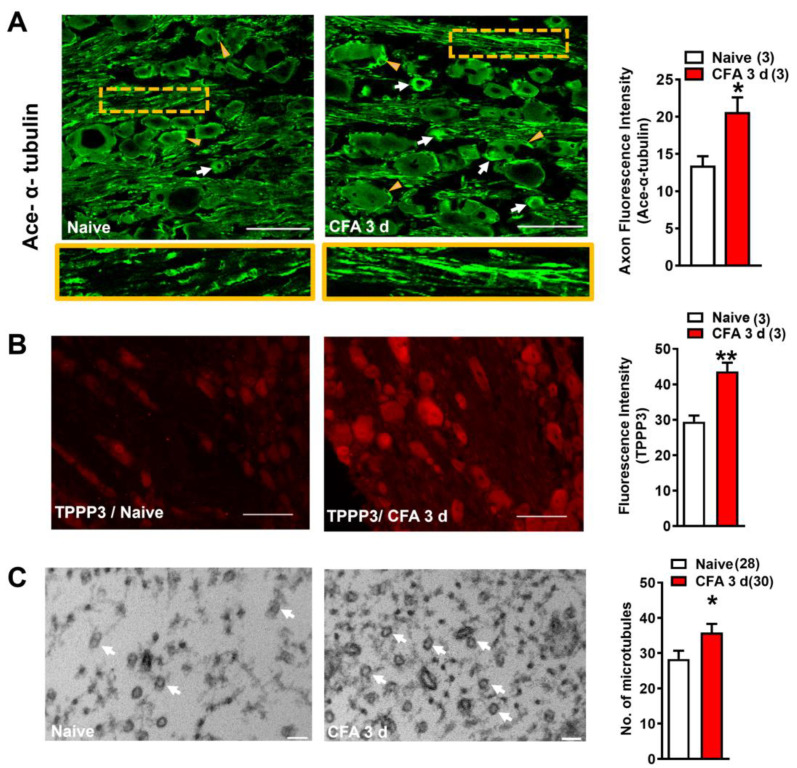
Polymerization of microtubules in rat DRG increased in the CFA-induced inflammatory pain. (**A**) Immunofluorescence staining showed a moderate increase of ace-α-tubulin in small-diameter DRG neurons at 3 d after CFA injection compared with that of the naïve (white arrows). While the ace-α-tubulin fluorescence intensity of axons showed a significant increase. The lower panel is the high magnification of the yellow boxed area in the upper panel. (**B**) The immunofluorescence staining demonstrated that the TPPP3 expression in DRG neurons at CFA 3 d was significantly increased compared with that of the naïve. (**C**) The number of microtubules examined by electronic microscopy significantly increased in unmyelinated or thin myelinated axons of DRG neurons at 3 d after CFA injection, compared with the naïve. All data were expressed as mean ± SEM (*n* = 3, 3 in (**A**,**B**); *n* = 28, 30 in (**C**)). Unpaired *t*-test, * *p* < 0.05, ** *p* < 0.01. Scale bar in (**A**,**B**): 100 µm. Scale bar in (**C**): 50 nm. Three independent experiments were repeated.

Our earlier study has revealed that Nogo-A in the DRG neuron promotes CFA-induced inflammatory pain [5]. The Nogo-A protein in DRG neurons was dramatically elevated after CFA administration (Supplementary Figure S1A,B). Knockout of Nogo-A significantly attenuated the inflammatory heat hyperalgesia, which was most prominent at 72 h after CFA administration (*n* = 5, WT; *n* = 9, KO; ** *p* < 0.01) (Supplementary Figure S1C,D). In this study, we hypothesized that the polymerization of microtubule modulated by Nogo-A in DRG neurons underlies the mechanism of inflammatory pain. Immunofluorescence staining demonstrated that both the satellite glial cells (yellow arrowheads) and the DRG neurons (white arrows), especially the small-sized ones, showed obvious acetylated α-tubulin immunoreactivity (Figure 1A). Although there was no obvious change in the overall immunoreactivity of acetylated α-tubulin in the DRG neuron 3 days after CFA injection, a significant increase in the axons in the CFA group was detected when compared with the naïve group (boxed areas in Figure 1A). Quantitative analysis demonstrated that there was a significant difference in the fluorescence intensity within axons between the two groups (*n* = 3, * *p* < 0.05, Figure 1A). In addition, the tubulin polymerization promoting protein 3 (TPPP3), which was shown to promote microtubule polymerization [32], was increased significantly in the DRG neurons 3 days after CFA injection (*n* = 3, ** *p* < 0.01) (Figure 1B), providing further support to the increased activity of polymerization during inflammatory pain. Furthermore, electron microscopy demonstrated that the number of microtubules in unmyelinated or thin myelinated sensory fibers of DRG neurons was obviously increased in the CFA group when compared with the naïve control (*n* = 3, * *p* < 0.05) (Figure 1C), lending further support to the elevated level of polymerization of microtubule during CFA-induced inflammatory pain.

### 2.2. Nogo-A Protein in DRG Promotes Inflammatory Heat Hyperalgesia by Increasing Microtubule Polymerization

**Figure 2:**
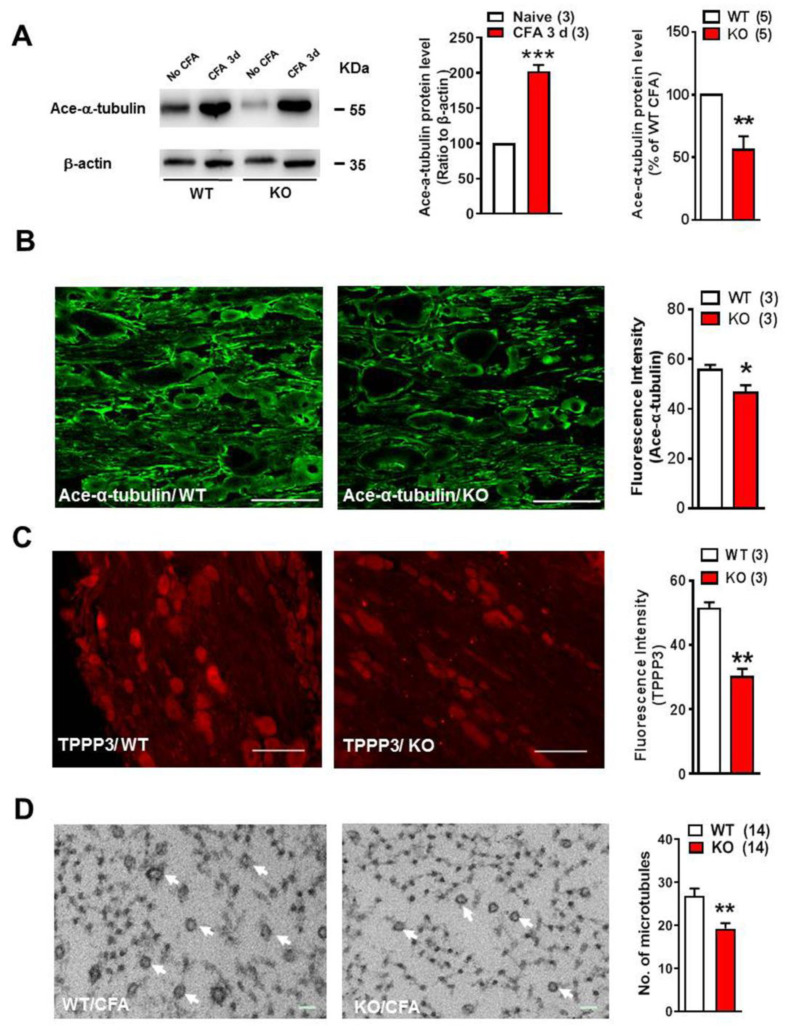
Nogo-A in DRG neurons promoted the polymerization of microtubules in the inflammatory heat hyperalgesia. (**A**) Western blot showed a significant down-regulation of ace-α-tubulin expression in DRG of Nogo-A KO rats at CFA 3 d, compared with that of WT. While an obvious increase of ace-α-tubulin expression in DRG at CFA 3 d in both WT and KO rats was detected. (**B**) The result of immunofluorescence staining also showed the decrease of the ace-α-tubulin in DRG in the Nogo-A KO rat with CFA administration. (**C**) Similarly, there was a significant decrease in TPPP3 fluorescence intensity in Nogo-A KO rats, compared with WT. (**D**) The result of electronic microscopy showed the number of microtubules in DRG neuronal axons of Nogo-A KO rats with CFA injection significantly decreased. * *p* < 0.05, ** *p* < 0.01, *** *p* < 0.001; npaired *t*-test (*n* = 3, 5, 3, 3, 14 respectively). Scale bar in (**B**,**C**): 100 µm. Scale bar in (**D**): 50 nm.

Western blotting showed that the CFA administration consistently elevated the expression of acetylated α-tubulin in DRG of WT rats (*n* = 3, *** *p* < 0.001) (Figure 2A). The acetylated α-tubulin was down-regulated in Nogo-A KO rats under inflammatory pain when compared with that in the WT (*n* = 5, ** *p* < 0.01), although the CFA injection also increased the level of acetylated α-tubulin in DRG of Nogo-A KO rats. (Figure 2A). Immunohistochemical study showed that the fluorescence intensity of acetylated α-tubulin in DRG sections with CFA administration was decreased in Nogo-A KO rats when compared with WT (*n* = 3, * *p* < 0.05) (Figure 2B). The level of TPPP3 protein was also reduced in KO rats 3 days after CFA treatment (*n* = 3, ** *p* < 0.01) (Figure 2C). The reduction of microtubules in DRG neurons in Nogo-A KO rats was further confirmed using electron microscopy. The microtubule number in unmyelinated or thin myelinated fibers of DRG was significantly reduced in Nogo-A KO rats with CFA injection when compared with that of WT rats (*n* = 3, ** *p* < 0.01) (Figure 2D).

### 2.3. pCRMP2 Was Increased in DRG in Nogo-A KO Rats after CFA Administration 

**Figure 3:**
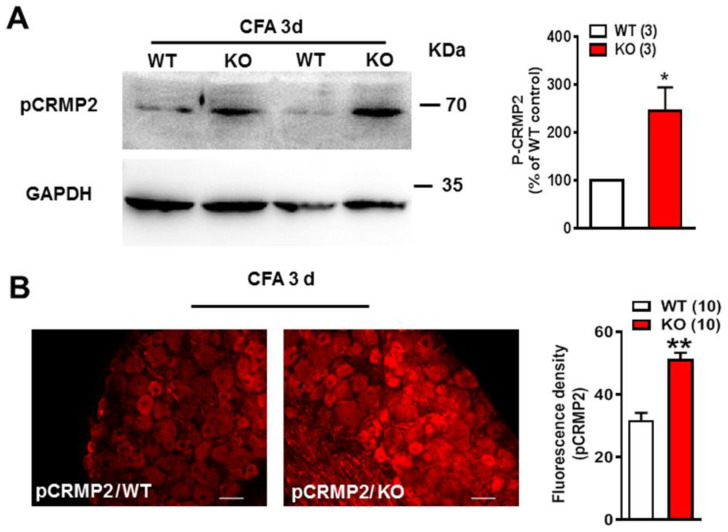
Nogo-A inhibited the expression of pCRMP2 in DRG of rats with CFA injection. (**A**) Compared with the WT, KO of Nogo-A increased the expression of pCRMP2 in rat DRG 3 days after CFA injection. * *p* < 0.05, unpaired *t*-test, *n* = 3. (**B**) The fluorescence density of pCRMP2 was obviously increased in DRG of Nogo-A KO rat after CFA 3 d, compared with WT. ** *p* < 0.01, unpaired *t*-test, *n* = 10. The scale bar is 50 μm. Data are expressed as mean ± SEM.

The phosphorylated form of CRMP2 (pCRMP2), which is shown to suppress microtubule assembly [31], was investigated in Nogo-A KO rats. Western blot results showed that the level of pCRMP2 was increased significantly in Nogo-A KO DRG when compared with WT 3 days after CFA treatment (*n* = 3, * *p* < 0.05) (Figure 3A). This increase in pCRMP2 was confirmed by immunohistochemistry, which showed a consistent and sustained increase in staining for pCRMP2 in Nogo-A KO DRG 3 days after CFA injection (*n* = 10, ** *p* < 0.01) (Figure 3B). These results indicate that Nogo-A in DRG neurons inhibits the phosphorylation of CRMP2, which promotes the polymerization of microtubules during inflammatory pain.

### 2.4. Nogo-A Was Necessary to Maintain Colocalization of Acetylated Microtubules and TRPV1

**Figure 4:**
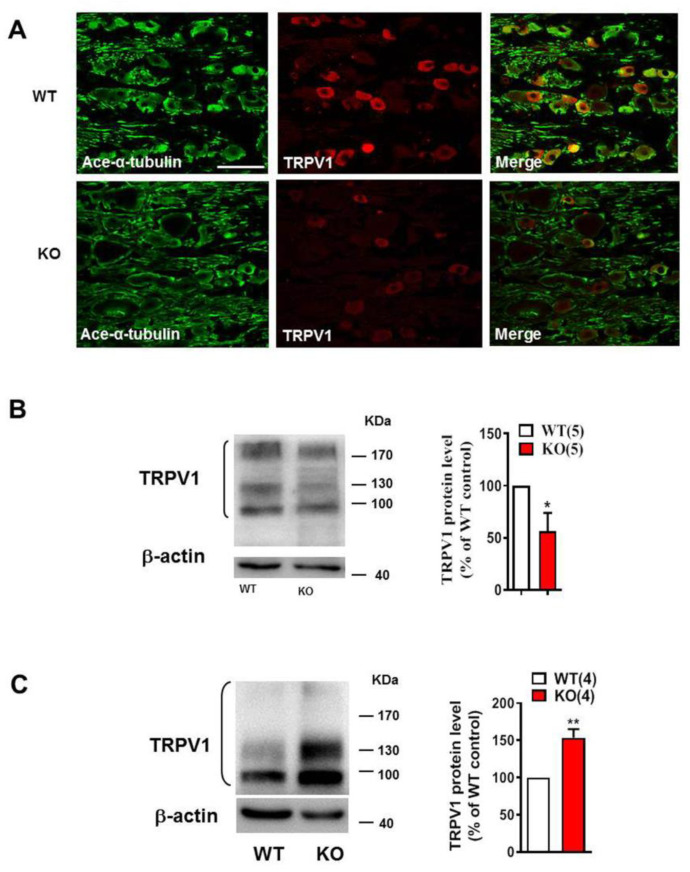
KO of Nogo-A decreased the co-localization of TRPV1 with ace-α-tubulin in the CFA- induced inflammatory pain. (**A**) The co-location of ace-α-tubulin and TRPV1 was decreased in the DRG neurons of Nogo-A KO with CFA injection. (**B**) The total TRPV1 (the monomer, glycosylation, and polymerization of TRPV-1) were included for the quantification and it was significantly decreased in DRG of Nogo-A KO rat with CFA injection, compared with that of the WT rat (* *p* < 0.05, unpaired *t*-test, *n* = 5). (**C**) The total TRPV1 was significantly higher in Nogo-A KO rat with CFA injection than that of WT after intrathecal injection of 10 μg paclitaxel, indicating that increase of the polymerization microtubule with paclitaxel could compensate the TRPV1 decrease resulted from the disassembly of microtubule due to KO of Nogo-A (** *p* < 0.01, unpaired *t*-test, *n* = 4). The scale bar in (**A**) is 100 μm.

Immunofluorescence staining showed that acetylated α-tubulin was expressed virtually in all DRG neurons and their axons in WT rats, whereas TRPV1 was observed only in a subpopulation of neurons (Figure 4A). The absence of Nogo-A in KO rats caused a reduction in the colocalization of acetylated α-tubulin and TRPV1, both in terms of the number of double-labeled cells and staining intensity. Western blot analysis demonstrated that knockout of Nogo-A significantly down-regulated the TRPV1 in DRG neurons of rats with CFA injection (*n* = 5, * *p* < 0.05) (Figure 4B). This down-regulation of TRPV1, resulted from the loss of Nogo-A, was rescued by intrathecal injection of paclitaxel (10 μg), which stabilizes the microtubules. It was found that the level of TRPV1 protein in Nogo-A KO rats was significantly higher than that of WT rats after intrathecal injection of paclitaxel (*n* = 4, ** *p* < 0.05) (Figure 4C), indicating that microtubule stabilization counters the effect of knockout of Nogo-A to TRPV1. Thus, we argued that the depolymerization of microtubules caused by KO of Nogo-A results in a reduced expression of TRPV1, which contributes to the analgesic effect in Nogo-A KO animals.

### 2.5. Inhibition of Microtubule Polymerization Attenuated Both CFA Induced Inflammatory Heat Hypersensitivity and Mechanical Pain in Spared Nerve Injury

**Figure 5:**
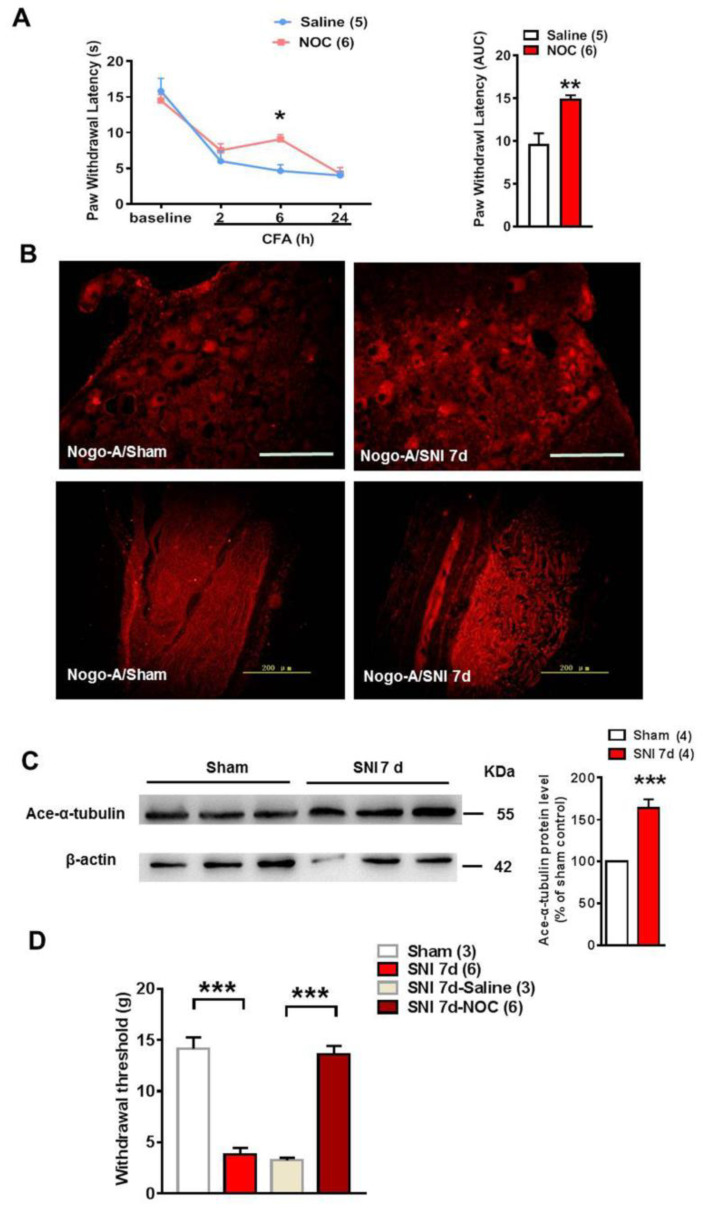
Inhibition of microtubule polymerization with nocodazole (NOC) attenuated significantly the CFA-induced inflammatory heat hyperalgesia and the mechanical pain in SNI. (**A**) Pre-intrathecal injection of 3.0 mg/kg NOC significantly elevated the PWL of rats after CFA administration. Time course of PWL and quantification analysis of the area under the curve (2–24 h) showed knock out of Nogo-A attenuated inflammatory heat hyperalgesia significantly (* *p* < 0.05, 2-way ANOVA followed by Bonferroni’s post hoc test, *n* = 5,6; ** *p* < 0.01, unpaired *t*-test, *n* = 5, 6). (**B**) Immunofluorescence staining showed that Nogo-A in DRG neuron (the up panel) and the sciatic nerve (the low panel) was both increased dramatically in SNI animal model. The scale bar is 100 μm and 200 μm, respectively. (**C**) The ace-α-tubulin protein expression was increased after SNI 7 d, compared with the sham (*** *p* < 0.001, unpaired *t*-test, *n* = 4). (**D**) The withdrawal threshold was decreased in SNI 7 d group, compared with the sham (*** *p* < 0.001, unpaired *t*-test, *n* = 3, 6), but after the nocodazole injection, the withdrawal threshold was significantly increased, compared with the SNI-Saline (*** *p* < 0.001, unpaired *t*-test, *n* = 3, 6). Data are expressed as mean ± SEM. The scale bars in (**B**) are 100 μm (up panel) and 200 μm (down panel) respectively.

To further confirm our hypothesis that polymerization of microtubules promotes inflammatory heat hyperalgesia, nocodazole (a microtubule destabilizer) was injected intrathecally before administration of CFA to the hind paw. It was found that inhibition of microtubule polymerization with nocodazole could elevate transiently the PWL at 6 h after CFA injection when compared with the control (*n* = 5, Saline; *n* = 6, Nocodazole, ** *p* < 0.01) (Figure 5A). In the model of spared nerve injury (SNI), the distribution of Nogo-A was increased in both the DRG neuron and sciatic nerve at SNI 7 days compared with the sham rat (Figure 5B). It was also found that the level of acetylated α-tubulin was increased significantly 7 days after induction of SNI (*n* = 4, *** *p* < 0.001) (Figure 5C). Furthermore, the withdrawal threshold was reduced significantly 7 days after SNI, but was increased dramatically 2 h after intrathecal administration of nocodazole (2 nmol) (Figure 5D), suggesting that microtubule polymerization is involved in the neuropathic pain in SNI (*n* = 3, Sham; *n* = 6, SNI 7 d; *n* = 3, SNI-Saline; *n* = 6, SNI-NOC; *** *p* < 0.001).

## 3. Discussion

The function of microtubules in pain modulation is elusive. The heterodimer formed by α-tubulin and β-tubulin is the basic building unit of microtubule [9]. Microtubules are always in the process of growth and collapse, which are termed “dynamic instability”. Since the acetylation of microtubules occurs only in polymerized microtubules, it was used as an indicator of polymerization in microtubules [17]. In this study, we found the increase of polymerized microtubules promoted by Nogo-A may contribute to the CFA-induced inflammatory pain. 

Nogo-A is a major subfamily of Nogo protein. It functions by modulating the cytoskeleton system after binding to its receptor complexes and activating various downstream signaling pathways [23]. Among these signaling pathways, Nogo-A is known to promote microtubule polymerization by modulating phosphorylation of CRMP2. The phosphorylated CRMP2 is shown to cause a loss of activity to polymerize microtubules [33]. In the current study, it was found that the heat hyperalgesia in rats with CFA administration was attenuated significantly in Nogo-A KO rats. And the underlying mechanism partially is that Nogo-A promotes microtubule polymerization in DRG through the inhibition of phosphorylation of CRMP2 during CFA-induced inflammatory pain. 

TRPV1 is one of the key molecules underlying inflammatory heat hyperalgesia, which integrates the chemical and thermal stimulation signals that induce pain [34]. Our laboratory has reported that Nogo-A in DRG neurons is necessary to maintain the function of TRPV1 in inflammatory pain [5]. In the current study, it was found that TRPV1 was co-localized with acetylated microtubules and the co-localization was reduced in Nogo-A KO rats. Furthermore, intrathecal injection of paclitaxel to stabilize the polymerized microtubules reversed the down-regulation of TRPV1 caused by the deletion of Nogo-A. These results suggested that the polymerization of microtubules mediated by Nogo-A is one of the major mechanisms regulating TRPV1 function in inflammatory pain. Intrathecal injection of nocodazole to destabilize the microtubules attenuated the CFA-induced inflammatory heat hyperalgesia, supporting that the polymerization of microtubules is necessary for the development of inflammatory pain.

Previous reports have already provided some indications on the interaction of tubulin and TRPV1. TRPV1 has multiple tubulin-binding sites [20]. The functions of TRPV1 are regulated by the TRPV1-tubulin complex on the membrane and sub-membranous regions in chemotherapy-induced peripheral neuropathy [19]. However, little is known about the functions of TRPV1-tubulin complex and its upstream modulator in pain. Here, we demonstrated that Nogo-A in DRG neurons promotes the polymerization of microtubules, which enhances the expression of TRPV1 and contributes to the development of inflammatory heat hyperalgesia. 

In our previous report, we showed that Nogo-A sustains the TRPV1 function in inflammatory pain via enhancing the polymerization of F-actin [5]. Here we showed further that Nogo-A contributes to inflammatory pain by promoting the stability of microtubules. Nogo-A was shown to modulate different components of cytoskeletons, including both F-actin and tubulin [23,24,26]. Moreover, both F-actin and tubulin are reported to modulate TRPV1 function [6,19]. Another Nogo isoform, Nogo-B, was shown to influence the function of vascular smooth muscle cells by modulating microtubule and actin dynamics [35]. Furthermore, the phosphorylation of Ser 824 residue on TRPV4 regulates its interaction with both F-actin and microtubules in a complementary manner [36]. These findings support that Nogo-A may influence both actin and tubulin simultaneously in the generation of inflammatory pain. 

Nogo-A, Nogo-B, and Nogo-C are the three Nogo isoforms encoded by the nogo gene [23]. All of them share one Nogo-66 functional domain at the C-terminus, whose function can be antagonized by the peptide antagonist, NEP1-40 [37]. Nogo-A is the longest isoform of Nogo subfamilies, which has at least three distinct functional domains, among which is the Nogo-A specific domain [38]. It was reported that Nogo-66 inhibits microtubule assembly in the injured axons via inducing phosphorylation of CRMP2 [26]; however, our results showed that instead of destabilizing tubulins, Nogo-A promotes polymerization of microtubules in DRG in inflammatory pain. These findings are consistent with that Nogo-A (Nogo-22 domain) promotes the activity of CRMP2 and mediates its inhibition of axon regeneration [29]. Similarly, RTN4 (Nogo) knockdown results in the destabilization of tubulins in cancer cells [28], indicating that Nogo promotes tubulin stability in cancer cells. This discrepancy suggested that different functional domains and isoforms of Nogo protein might play different roles for the tubulin stability into diverse pathological processes. The Nogo-A KO rats used in this study have the amino acid sequences with the exon 3 deleted, which is not shared with the other Nogo isoforms. In future experiments, we should specify the Nogo-A specific region that promotes the polymerization of microtubules. 

The previous study has shown that acetylated microtubules are involved in itching and tactile signaling [18]. Our experiment demonstrates that the polymerization of microtubules contributes to the CFA-induced inflammatory pain, and the disassembly of the microtubules with nocodazole attenuates inflammatory heat hyperalgesia. Furthermore, we have demonstrated that microtubules also participate in the mechanical pain of the SNI model. The Nogo-A expression and the acetylated α-tubulin in DRG of rats with SNI is significantly increased 7 d after injury. Similarly, intrathecal injection of nocodazole attenuates the mechanical pain. Hence the polymerization of microtubules might be a common mechanism underlying different types of pain. Depolymerization of the microtubules in the pain process might be a possible target for developing novel analgesic drugs. Of course, further study is needed to confirm this hypothesis.

**Figure 6:**
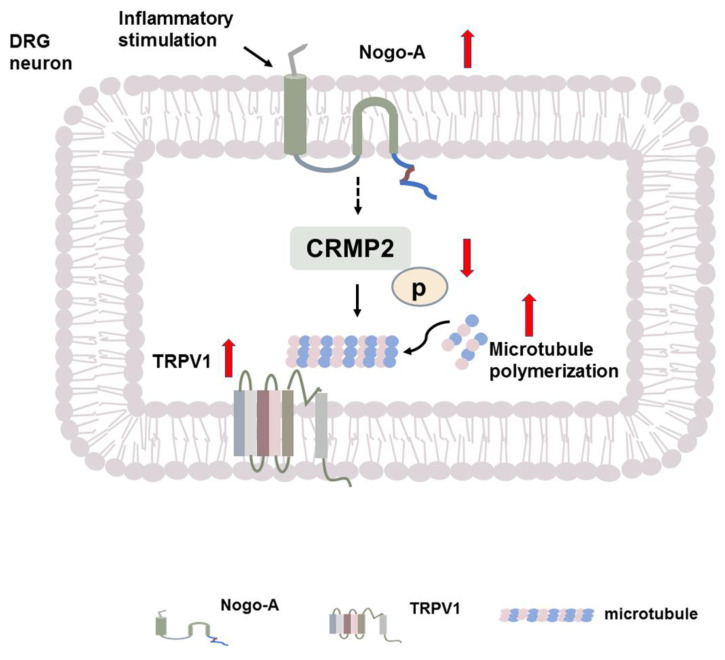
A model for the regulation of microtubule by Nogo-A in inflammatory heat hyperalgesia. The inflammatory stimulation elevated the Nogo-A expression in DRG neurons in the CFA-induced inflammatory pain [5]. Nogo-A in DRG neuron inhibits the phosphorylation of CRMP2 and increases the activity of CRMP2. The active CRMP2 enhances the polymerization of microtubules, which supports the function of TRPV1 and contributes to the development of inflammatory pain.

In summary (Figure 6), our results support that the increase of Nogo-A in DRG neurons promotes microtubule polymerization via inhibiting the phosphorylation of CRMP2 in CFA-induced inflammatory pain. Polymerization of microtubules is necessary for maintaining the function of TRPV1 and the development of CFA-induced inflammatory heat hyperalgesia. In addition, microtubule polymerization also promotes the development of mechanical pain in SNI. 

## 4. Materials and Methods

### 4.1. Animals

Male Sprague-Dawley rats weighing 200–250 μg were supplied by the Animal Center of Peking University Health Science Center. They were housed in temperature-controlled rooms on a 12 h/12 h light-dark cycle (lights on 8 AM) with free access to food and water. All experimental procedures were approved by the Animal Care and Use Committee of Peking University, and efforts were made to minimize the discomfort of the animals.

The Nogo-A (Rtn4) KO rats were generated by Nanjing Biomedical Research Institute of Nanjing University (Nanjing, China). Nogo-A (Rtn4)–KO rats were generated by targeting the sequence of Nogo-A (Rtn4)-201 transcript using the clustered regularly interspaced short palindromic repeats (CRISPR)/CRISPR-associated (Cas) 9 system. The success of knockout of Nogo-A in rats was confirmed by Western blot and immunostaining [5].

### 4.2. Inflammatory Pain Model

The complete Freund’s adjuvant (CFA) (F5881; Millipore Sigma, Burlington, MA, USA) was subcutaneously injected into the left hind paw to induce chronic inflammatory pain. The rats were anesthetized with isoflurane inhalation. The left hind paw and surrounding skin were sterilized with 75% alcohol and then injected with 100 µL of 25% CFA diluted in incomplete Freund’s adjuvant (F5506; Millipore Sigma, Burlington, MA, USA) to avoid spontaneous pain behavior. The CFA injection produces local swelling of the hind paw characterized by erythema, edema, and hypersensitivity. The animals exhibited normal grooming behaviors and weight gained during the whole course of the experiment.

### 4.3. Spared Nerve Injury (SNI) Model

SNI model was generated according to a previous report [39]. The rats were anesthetized by isoflurane inhalation. The skin was incised at the upper margin of the hind limb in the prone position, and the muscles were separated to expose the sciatic nerve and its branches. The tibial nerve and common peroneal nerve were ligated and cut, and the sural nerve was preserved without injury. The wounds were closed by sutures. In the sham-operated group, the sciatic nerve and its branches were exposed but without ligation. The rest of the procedures were the same as those in the experimental groups. 

### 4.4. Behavior Test

#### 4.4.1. Assessment of Heat Hypersensitivity 

The methods of thermal hyperalgesia assessment were adopted from a previous study [40]. Before the test, rats were placed on a glass plate and covered with a transparent plexiglass cover (18 cm × 8 cm × 8 cm) for 20 min for adaptation to the environment. During the test, the radiant heat lamp was focused on the central part of the hind paw to measure the paw withdrawal latency (PWL). The intensity of radiant heat was adjusted so that the PWL of the basic state of the rat is 10–15 s. The measurements were repeated 5 times for each rat with each test interval of 15 min. All behavioral tests were carried out in a blinded fashion. 

#### 4.4.2. Assessment of Mechanical Allodynia

The von Frey test was used to test the mechanical allodynia in rats with SNI. Briefly, von Frey filaments (Danmic Global LLC, San Jose, CA, USA) were used to measure the 50% paw withdrawal threshold. A series of filaments were applied to the plantar surface of the left hind paw with pressure to buckle them in a consecutive sequence, starting with the filament that had a buckling weight of 2 g. Withdrawal of the hind paw was recorded as a positive response, whereas the absence of paw lifting within 5 s indicated a negative response. The median 50% threshold (g) was calculated according to the up-down method [41].

### 4.5. Intrathecal Injection of Nocodazole or Paclitaxel

The method of subarachnoid cannula injection was described in our previous report [5]. The rats were anesthetized by inhalation of isoflurane. Hairs were removed from the back and the skin was sterilized. The intraspinal space between lumbar vertebra 4 and 5 (L4–L5) was chosen as the site for the insertion of a needle. Polyethylene-10 catheters (outer diameter, 0.61 mm) were implanted with a catheter-through-needle technique to reach the lumbar enlargement of the spinal cord. The correct intrathecal location was confirmed by tail-flick or paw retraction. The outer end of the catheter was plugged and fixed to the skin upon closure of the wound. After the operation, the rats were housed alone. Seven days after surgery, the rats were injected with nocodazole (3.0 mg/kg) [42] or with the same amount of saline. After half an hour, 100 μL 25% CFA was injected into the hind paw of each rat. The PWL was recorded at 2 h, 6 h, and 24 h. In rats with SNI, the same amount of nocodazole was intrathecally injected to test the mechanical allodynia by von Frey test. In another experiment, 10 μg paclitaxel was intrathecally injected a half-hour after the CFA injection. And 6 h later, the DRG of L4 and L5 were removed to examine the TRPV1 protein in wild type (WT) and Nogo-A knock-out (KO) rats.

### 4.6. Western Blot Analysis

All rats were deeply anesthetized with 10% chloral hydrate (0.3 g/kg, i.p.). The nitrocellulose membranes were incubated overnight at 4 °C with the primary antibody. The following commercially available antibodies were used as primary antibodies: ace-α-tubulin (1:10,000, sc23950, Santa Cruz Biotechnology, Dallas, Texas, USA), pCRMP2 T514 polyclonal antibody (1:1000, ab62478; Abcam, Waltham, MA, USA) guinea pig anti-TRPV-1 polyclonal antibody (1:1000, AB5566; MilliporeSigma, Burlington, MA, USA), GADPH (1:2000, TA-08, Zhongshan Golden Bridge Biotechnology, Guangdong, China), and β-actin (1:2000, TA-09, Zhongshan Golden Bridge Biotechnology, Guangdong, China). The blots were washed 3 times in Tris-buffered saline-Tween. The membranes were incubated with horseradish–conjugated secondary antibody (1:2000, goat anti-rabbit, rabbit anti-goat, rabbit anti-guinea pig, or goat anti-mouse; Bio-Rad, Hercules, CA, USA) for 1 h at room temperature, and developed with a chemiluminescence kit (sc-2048, Santa Cruz Biotechnology). 

### 4.7. Immunofluorescence Staining

Sections of DRG or sciatic nerve from WT or Nogo-A KO rats, with or without CFA or SNI model, were used in this experiment. Sections of embedded tissue were collected using a cryostat, dried, and hydrated with PBS for 20–30 min at room temperature. 0.3% Triton x-100 in PBS was used for perforation at room temperature for 20 min. Sections were washed 3 times with PBS and incubated in the primary antibody overnight at 4 °C. The slides were washed with PBS and incubated with the fluorescent secondary antibody or dye at room temperature for 1 h or stored at 4 °C overnight. The primary antibody used: ace-α-tubulin (1:10,000, sc23950; Santa Cruz Biotechnology), pCRMP2 T514 polyclonal antibody (1:1000, ab62478; Abcam), guinea pig anti-TRPV-1 polyclonal antibody (AB5566; MilliporeSigma, Burlington, MA, USA), and TPPP3 (1:1000, bs-17172R, Bioss, Massachusetts, USA). The corresponding secondary antibodies for ace-α-tubulin, TRPV1, TPPP3, and pCRMP2 were FITC-conjugated goat anti-mouse IgG (1:200; Jackson ImmunoResearch, West Grove, PA, USA), TRITC-conjugated goat anti-guinea pig IgG (1:200, Thermo Fisher Scientific, MA, USA), TRITC-conjugated goat anti-mouse IgG (1:200, Jackson ImmunoResearch, West Grove, PA, USA), TRITC-conjugated donkey anti-rabbit IgG (1:200; Thermo Fisher Scientific, MA, USA), respectively. 

### 4.8. Transmission Electron Microscopy

Transcardial perfusion was performed in rats after anesthesia with chloral hydrate, followed by rapid perfusion of 250 mL normal saline at 37 °C, and slow perfusion of 200~250 mL fixative mixture of 4% paraformaldehyde and 1% glutaraldehyde. DRG on both sides at L4–L5 were quickly collected and post-fixed with 1% osmium for 1.5 h at 4 °C. The tissues were then dehydrated in acetone and embedded in resin. Ultrathin sections of the DRG were obtained. In the cross-section of axons, the number of microtubes in unmyelinated or thin myelinated axons within a defined area (1.2 μm^2^) was counted in axons with a diameter less than 5 m, which consist of fibers responsible largely for the inflammatory heat sensitivity [43], using an electron microscope (JEOL, JEM-1400 Plus). 

### 4.9. Statistical Analysis

All data are expressed as mean ± SEM. Statistical analyses were performed using GraphPad Prism software 7.0. Comparisons between experimental and control groups were made by using the Student’s t-test, One-way ANOVA followed by Newman–Keuls post hoc test, or 2-way ANOVA followed by Bonferroni’s post hoc test. Statistical significance was set at *p* < 0.05. For Western blot and immunofluorescence imaging, the intensity of relevant bands and pictures were measured with ImageJ software (National Institutes Health, Bethesda, MD, USA). The *n* number in the electron microscopy experiment referred to the number of the defined area counted, while it was the animal number in the other experiments.

## Data Availability

Not applicable.

## Supplementary Materials

The following are available online at https://www.mdpi.com/article/10.3390/ijms221910360/s1, Figure S1: Nogo-A in DRG contributed the development of CFA-induced inflammatory pain.
