# Supplementary material for: Nogo-A Induced Polymerization of Microtubule Is Involved in the Inflammatory Heat Hyperalgesia in Rat Dorsal Root Ganglion Neurons
Source: Int J Mol Sci. 2021 Sep 26;22(19):10360. doi: 10.3390/ijms221910360 (PMC8508904; doi:10.3390/ijms221910360)
Supplement: Supplementary file 1 [file ijms-22-10360-s001.zip › ijms-1353651-supplementary.pdf]

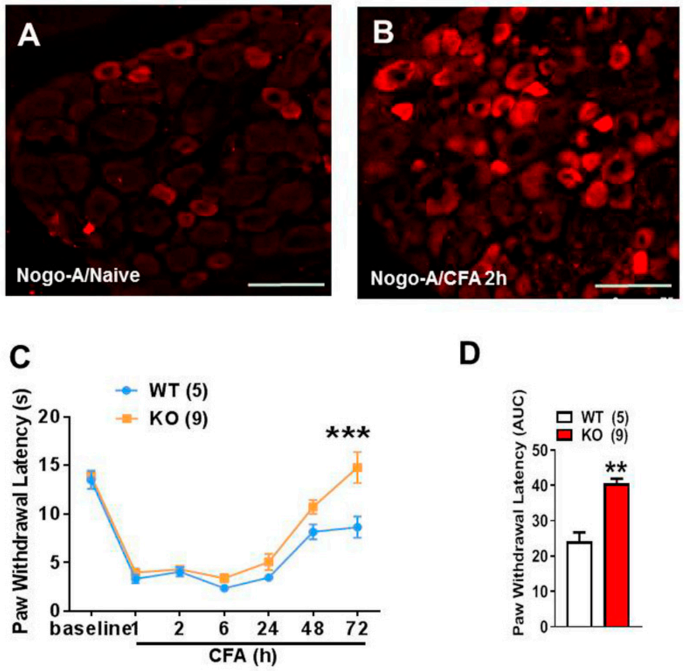

Supplementary Figure S1. Nogo-A in DRG contributed the development of CFA-induced inflammatory pain. (A,B) Immunofluorescence staining demonstrated that the Nogo-A expression in DRG neurons of CFA-treated rats (B) was obviously elevated compared with that of the naive rats(A). (C,D) Time course of PWL and quantification analysis of the area under the curve (1－72 h) showed knock out of Nogo-A attenuated the inflammatory heat hyperalgesia significantly after CFA administration (*** $p < 0.001$, 2-way ANOVA followed by Bonferroni’s post hoc test, n = 5, 9; ** $p < 0.01$, unpaired t-test, n = 5, 9).
